# Chelating effect in short polymers for the design of bidentate binders of increased affinity and selectivity

**DOI:** 10.1038/srep15633

**Published:** 2015-10-26

**Authors:** Sara Fortuna, Federico Fogolari, Giacinto Scoles

**Affiliations:** 1 MOlecular NAnotechnology for LIfe Science Applications Theory Group, Department of Medical and Biological Sciences, University of Udine, Italy; 2 Department of Biology, Temple University, Philadelphia (PA), USA

## Abstract

The design of new strong and selective binders is a key step towards the development of new sensing devices and effective drugs. Both affinity and selectivity can be increased through chelation and here we theoretically explore the possibility of coupling two binders through a flexible linker. We prove the enhanced ability of double binders of keeping their target with a simple model where a polymer composed by hard spheres interacts with a spherical macromolecule, such as a protein, through two sticky spots. By Monte Carlo simulations and thermodynamic integration we show the chelating effect to hold for coupling polymers whose radius of gyration is comparable to size of the chelated particle. We show the binding free energy of flexible double binders to be higher than that of two single binders and to be maximized when the binding sites are at distances comparable to the mean free polymer end-to-end distance. The affinity of two coupled binders is therefore predicted to increase non linearly and in turn, by targeting two non-equivalent binding sites, this will lead to higher selectivity.

The ability of capturing target molecules with high affinity and selectivity is key for the development of new sensing devices, such as diagnostic tools and biosensors, and the design of side-effects free drugs. While binders for large organic molecules such as antibodies and their engineered fragments^1^ or DNAs and RNAs based aptamers^2^ are typically optimized either experimentally^3^ or computationally^4^ by generating, screening, and selecting the best candidate out of a large number of possibilities, a complementary approach consists in the design of polidentate binders.

Rigid polidentate binders are known to have enhanced affinity and selectivity compared to those of a collection of monodentate binders. The simplest example being that of dicarboxylic acids binding a metal with stronger affinity with respect to that of the corresponding uncoupled acids. Here the rigidity of the coupling scaffold guarantees little entropy variations upon binding. This characteristic, or chelating effect, can be scaled up to design binders with enhanced affinity towards organic molecules or proteins. For instance antibodies are capable of binding their target thanks to a number of coupled peptidic loops^5^, and rigid synthetic scaffolds such as calixarenes^6^ and porphyrins^7^ have been used to enhance the affinity of single peptides loops by coupling multiple loops together. The same consideration applies to multivalent nanoparticles and colloids capable of binding receptors coated surfaces^8^ and cells^9^,^10^. In drug design it is further known that the use of flexible moieties, such as polyethylene glycol chains, can enhance the stability and effectiveness of pharmaceuticals^11^,^12^. Flexible multivalent binders have been shown successful to enhance specificity and binding affinity for the immobilization of biomolecules on surfaces^13^ and extracellular matrix^14^ and it has been shown that the surface density of multivalent polymer increases faster than linearly with the surface density of binding sites^15^. It is also known that in systems where every monomer interacts with the substrate^16^,^17^, in the limit of an infinite number of interacting monomers, observables like the number of adsorbed monomers follow scaling relations typically found in phase transitions^17^. In the present system the number of interacting monomers remains two, regardless the length of the coupling polymer.

Indeed, coupling the binding moieties through long polymeric flexible linkers is advantageous due to their low cost, ease of synthesis, and large variety of structural and chemical properties such as solubility, hydrophobicity, reactivity they have. The huge variety of possibilities offered raises questions on whether and under which conditions flexible linkers would be as thermodynamically advantageous as their rigid counterparts, and which would be the conditions upon which the chelating effect prevails over entropic, confinement, and excluded volume effects^18^. While it is known that selectivity can be improved in multivalent nanoparticles by making their individual ligand-receptor bonds weaker^8^, that the surface assembled structure of thetered polymers largely depends on the spacer length^19^, and that weak spacer-receptor interactions, such as that of PEG, can enhance the binding^20^ of a single chain grafted to a surface^20^,^21^, it is yet unknown wether the chelating effect would hold for flexible chelating agents when capturing nanoparticles or macromolecules in solution.

Here we show that flexible linkers are convenient over single binders for the chelation of macromolecules, showing the chelating effect to hold for flexible linkers. From a theoretical point of view, the problem can be formulated as the study of the adsorption of a polymer with two sticky spots to a curved surface or a nanoparticle as schematized in Fig. 1a. In general, when discussing polymer adsorption on a curved surface, three behavioral regimes can be identified depending on the relative size between the polymer and the particle^22^: (i) the size of the nanoparticle is larger than the radius of gyration (*R*_g_) of the polymer (this has been widely studied in the context of polymer brushes, with MD simulations and coarse grained models), (ii) they are comparable, (iii) the size of the nanoparticle is far smaller (the so-called “protein limit”^23^). Only the latter case has been explored both analytically and with a 3D lattice model of a chain grafted through both its terminals^24^, while here we explore the regime in which *R*_g_ is comparable to the size of the spherical particle, a setup relevant for the design of real binders.

The chelation effect will be studied by non-equilibrium simulations at infinite dilution. A starting bound configuration will be allowed to reach a final unbound configuration through a finite number of intermediate states. While making clear that the statistical quantities are derived under non equilibrium simulations, we will discuss the results according to standard equilibrium thermodynamic language keeping in mind that the statistical quantities calculated over these states will then not be the thermodynamic ones but will be non-equilibrium quantities. Nevertheless the results obtained will highlight important effects in chelation, including the effect of the excluded volume, of the number of polymeric units between binding ends, and of the relative positions of binding patches on the target macromolecule.

## Model

**Figure 1:**
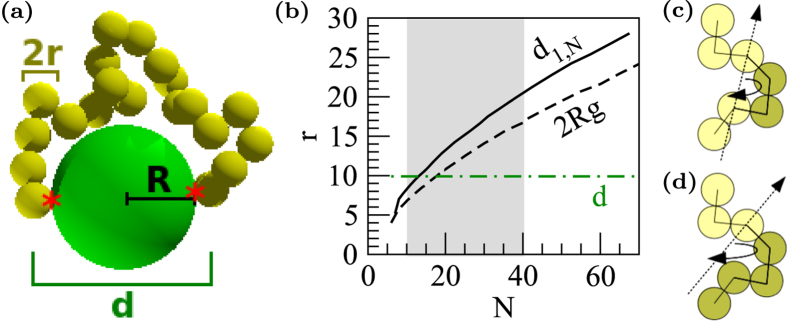
(**a**) The system is composed by a spherical target of radius *R* (large green sphere) with two binding sites (red asterisk) interacting with a polymeric chain composed by *N* equal beads with radius *r* (small yellow spheres). Only the extremes of the polymer can interact with the target binding sites. (**b**) Comparison between the free polymeric chain the end-to-end distance *d*_1,*N*_ (solid line), radius of gyration 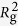 (dashed line) and the minimum distance *d* between its extremes when the system is bound (dashed dotted line) as a function of *N*. The range of *N* considered in this work is highlighted in gray. In the simulations, possible polymer moves are (**a**) krankshaft and (**b**) pivot move (the beads that rotate around the randomly selected axes are highlighted by a darker color).

In detail, we study by Monte Carlo simulations the transition between initial bound states and final unbound states of a finite polymeric chain, composed by *N* equal beads of radius *r*, interacting through its end-beads with a spherical target as shown in Fig. 1a. The beads interact with each other by a hard-sphere potential


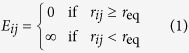


where *r*_*ij*_ is the distance between two beads and *r*_eq_ = 2*r*. The target is modeled as a hard sphere with radius *R* with two sticky spots on its surface separated by an angular distance *α*. The first and the last beads interact each with one of the two sticky spots with a Lennard-Jones potential of the form:


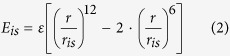


where *ε* is the depth of the well and *r*_*is*_ is the distance between a sticky spot and its respective bead, while the other beads interact with the protein with a hard-sphere potential of the same form as that of Eq. 1. In this notation *E*_1*s*_ and *E*_*Ns*_ are the binding energy between the first and the last beads with one sticky spot, respectively. All the energies are in units of *k*_B_*T*, where *k*_B_ is the Boltzmann constant and *T* the temperature. We define the following reduced units: T ^*^ = *k*_B_*T*/*ε* and E ^*^ = *E*/*ε*.

During the simulation the protein position is fixed while the polymer is allowed to perform Monte Carlo moves. A number of simulation schemes have been developed to ensure appropriate sampling in system where bond formation is important such as the topological Configuration Bias Monte Carlo for surface-adsorbed polyfunctionalized polymers^25^ or the aggregation-volume-bias Monte Carlo scheme for the simulation of strongly associated fluids^26^, but in this particular case the dilution and the simplicity of the system under investigation allows for a simple standard scheme. The polymer is allowed to perform both crankshaft and pivot moves (Fig. 1c,d). The former are performed by randomly selecting two beads defining a rotational axes about which contained beads are rotated by a random angle, the latter is performed by randomly selecting both a bead and random axes about which one of the two portion of the polymer will rotate. The probability with which one of the two possible moves is selected is tuned to obtain a uniform sampling over all the beads. The latter condition together with the random selection of the portions of the system to be moved guarantee detailed balance. At each simulation step either a pivot or a crankshaft move is attempted and the new configuration is then accepted or rejected following the Metropolis rule^27^. Every simulation consists of an equilibration phase followed by a production phase, both consisting of 2500*N* attempted moves which guarantee both the equilibration of the system and an adequate sampling of the conformational space. All the averages are calculated over 100 simulation runs.

We set the polymer beads radii to the average radius of an amino acid *r* = 0.38 nm and that of the target to the average radius of a globular protein *R* = 1.5 nm. The latter corresponds to a protein of 80 amino acids, following *R*_g_ = 2.2*N*^0.38^ 28 where 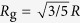. We consider polymers with *N* from 8 to 70, with particular focus on those whose radius of gyration 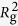 is comparable to the protein size (Fig. 1b). 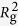 is defined as:


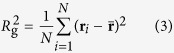


where 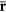 is the mean position of the beads.

## Results

### Order Parameters

**Figure 2:**
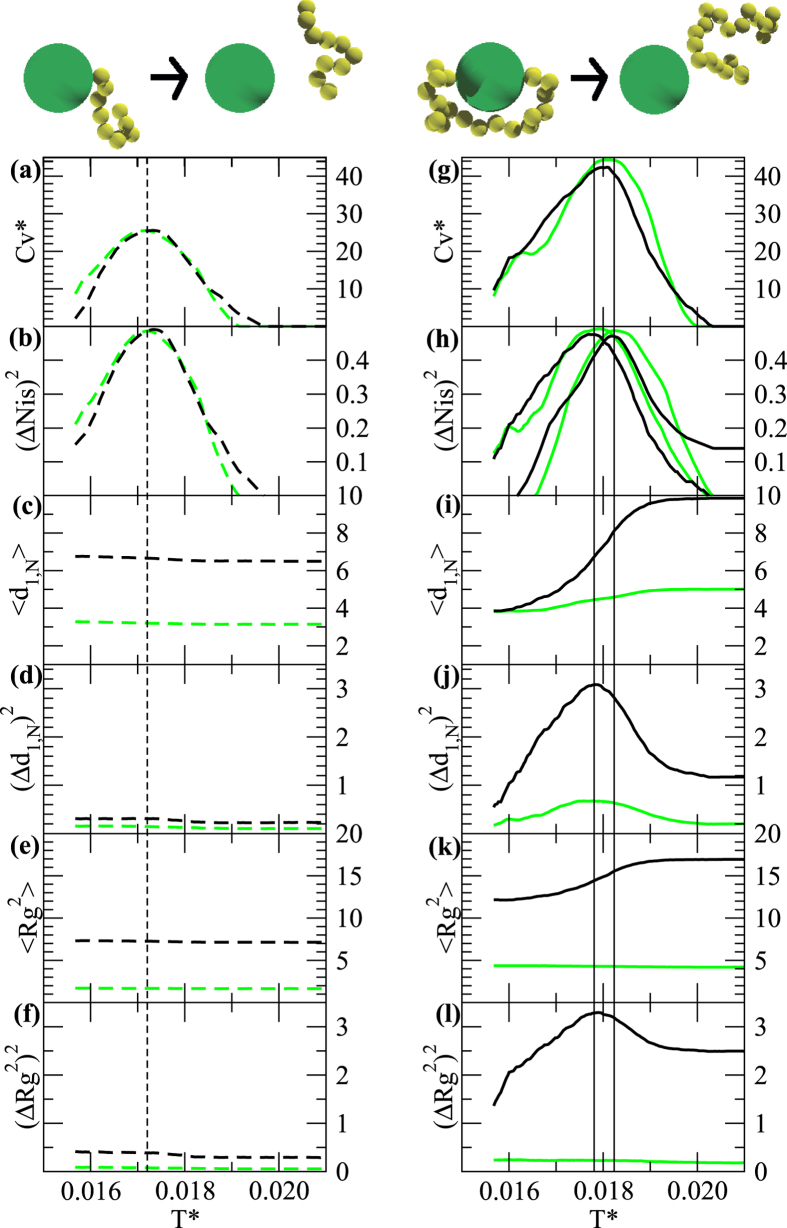
Snapshots and order parameters for single binders composed by 10 (gray/green dashed lines) and 30 (black dashed lines) beads and double binders of 20 (gray/green solid lines) and 60 beads (black solid lines) as a function of T ^*^. Vertical lines indicate the maximum of (Δ*N*_*is*_)^2^ for the *N* = 30 single binder (dashed line) and the *N* = 60 double binder (solid lines).

We compare the dissociations of the two systems schematized at the top of Fig. 2: (a) a polymer binding to the target with only one end, or monodentate binder, and (b) a polymer binding with both its ends to a system with binding sites located at its opposite sides, or bindentate binder. We run simulations by setting *ε* = 1. We build a chain of 200 simulations with *k*_B_*T* = [0.015, 0.025] where the last configuration of each temperature step is the starting configuration of the next. We identify bound/unbound transitions by calculating the fluctuation formula analogous to the specific heat at constant volume *C*_*V*_:


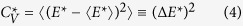


where E^*^ is the total energy of the system at a given simulation step, and 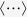 indicates an average taken over the production steps and over the 100 replicas of the same system.

During each simulation multiple binding/unbinding events occur. The bound/unbound transition is then characterized by introducing an order parameters based on the polymer-end/sticky spots distance:


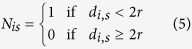


where *d*_*i*,*s*_ is the distance between the polymer first and last bead from their respective sticky spots. For double binders, to break the symmetry of the system and get an insights on the separate contributions of the two binders, the calculation of 〈Δ*N*_*is*_〉 is performed first by averaging over configurations, then by averaging over different runs by differentiating the statistics of the binders that detach first from that of the binders that detach last. These parameters together with 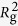 and the end-to-end distance *d*_1,*N*_ and their fluctuations, allow a throughout characterization of the chelating effect.

To characterize the chelating effect we compare single binders with *N* = 10, 30 (Fig. 2a–f) to the corresponding double binders with *N* = 20, 60 (Fig. 2g–l). Single binders show a peak in the 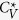 (Fig. 2a) at the same position of the peak in the (Δ*N*_*is*_)^2^ (Fig. 2b) corresponding to the polymer detachment, as highlighted by the vertical dashed line in Fig. 2. This is associated with a negligible variation (barely observable in the plot) of 〈*d*_1,*N*_〉 and 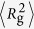 (Fig. 2c,e) and their fluctuations (Fig. 2d,e). On the other hand, for the double binders the 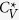 peaks between the maximum of the two (Δ*N*_*is*_)^2^ (see the vertical solid lines in Fig. 2). In this latter case, the behavior of 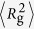 and 〈*d*_1,*N*_〉 depend on the polymer length. For *N* = 20 〈*d*_1,*N*_〉 changes by 25% (Fig. 2i), while 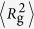 is constant (Fig. 2k). This is not true for *N* = 60 where 〈*d*_1,*N*_〉 almost doubles. Here the maximum of (Δ*d*_1,*N*_)^2^ (Fig. 2i) corresponds to the detachment of the first polymer-end (Fig. 2h) and to the maximum variation of 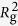 (Fig. 2k). Overall, single binders do not change 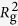 upon detaching, as well as the double binders with *R*_g_ ~ *R* such as *N* = 20.

**Figure 3:**
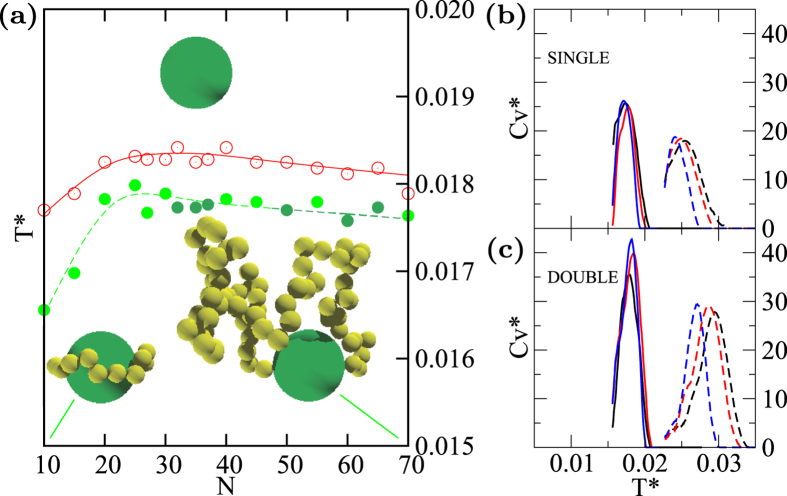
(**a**) max 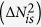 as a function of the polymer length for each end of a double binder (open and solid circles). Lines are cubic splines and a guide for the eye. 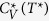 for (**b**) single and (**c**) double binders with (solid lines) and without (dashed lines) the excluded volume of the target for *N* = 10 (black), *N* = 25 (red), and *N* = 70 (blue). Running averages over 10 points.

The peaks in the (Δ*N*_*is*_)^2^, highlighted by the vertical lines in Fig. 2, also identify the transition temperatures associated with the bound/unbound transitions. For single binders that corresponds to T ^*^ = 0.017, while for double binders that depends on their lengths. In Fig. 3a the location of the maximum of (Δ*N*_*is*_)^2^ for double binders defines their phase diagram. In short chains (*N* = 10) one can easily detach one end while keeping the other fixed for a large temperature range. The onset of the detachment of the first end is at lowest T ^*^ than that of single binders. The detachment T ^*^ then increases and peaks at *N* = 25, then the curves monotonically approach lower T ^*^ with the elongation of the chain not having a strong effect on the stability of the chains. This shift of the transition temperature towards higher T ^*^ is consistent to the so-called “law of mass action”: coupled binders of appropriate length are favored at higher temperatures than single binders as they are kept locally concentrated thanks to the action of their partner.

Further, by running a set of simulations by switching off the beads-protein hard-sphere potential, for all cases the excluded volume effect is simply that of moving the transitions towards lower temperatures and bringing all the *C*_*V*_ curves closer to each other (Fig. 3b,c). As the peak of the *C*_*V*_ is associated with the adsorption of the polymer beads to their target, the target excluded volume contributes to destabilize the chelated system and the effect cannot be compensated by a simple change of *N*.

### Free Energies

To quantify the effect of chelation for flexible linkers we compare the dissociation free energies of double binders with that of two independent single binders of half-length. While on lattice models the free energy of confined polymers can be estimated by simply counting configurations, and when dealing with real systems enhanced methods such as umbrella sampling are often employed, for this system having only a single interaction we chose thermodynamic integration^29^. More precisely, we calculate the dissociation free energies associated with the non-equilibrium process of bringing a binder from a bound state to an infinite distance from the target. While an accurate estimation of this quantity would require integrating over the whole configurational space, a good approximation is to integrate along the detachment path. The latter can be defined by introducing a coupling parameters *λ* for tuning the interparticle potential. Tuning *λ* allows to sample polymer configurations at infinitesimal decreasing values of the interparticle potential. The polymer with non interacting energies is taken as a proxy for the unbound polymer. The sampled configurations define a possible detachment path. Once configurations along path are collected, it is then possible to recalculate the unbiased energies and integrate along the detachment path. Formally, we set the potential between the end beads of the polymer and the protein to *E*_*is*_(*λ*) = *λE*_*is*_ and we calculate the free energy difference between bound and unbound states as:


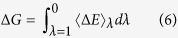


where Δ*E* = *E*_1*s*_(*λ* = 1) + *E*_*Ns*_(*λ* = 1) and 〈Δ*E*〉_*λ*_ is its ensemble average calculated over a set of configurations sampled at each selected values of *λ*. We further define ΔG^*^ = Δ*G*/*ε*. We perform thermodynamic integration at T ^*^ = 0.015, temperature at which all the systems are bound. The final result is then averaged over 100 possible paths.

**Figure 4:**
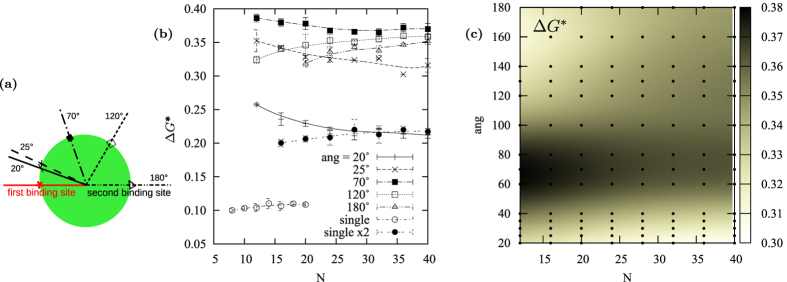
(**a**) Binding angles scheme. (**b**) ΔG^*^ as a function of the double binder length *N*. Each line corresponds to a different binding angle, as schematized in (**a**). For comparison also the ΔG^*^ of a single binder, and the ΔG^*^ of two single binders of equal length whose length sums up to *N*. Lines are Bézier curves and are only a guide for the eyes. (**c**) ΔG^*^ as a function of both the binding angle and the double binder length *N*. A darker color corresponds to more favorable ΔG^*^. Data points, highlighted by black dots, are interpolated with the gnuplot “gauss” smoothing kernel.

We compare the ΔG^*^ for dissociating two coupled binders as a function of *N* for binding angles ranging from 20° to 180° (schematized in Fig. 4a) with that of two single binders of length *N*/2. The ΔG^*^ is positive for all the explored cases (Fig. 4b) making unfavorable the detachment of both monodentate and bidentate polymers. Double binders, when the angle between the two binding sites is 20°, while being favored do not take advantage of the chelating effect for *N* > 30. The ΔG^*^ increases by increasing the binding angle reaching a maximum for all the explored chain lengths for angles between 60° and 80°, it then slowly decreases for larger binding angles. Constraining long polymers at small distances is unfavorable, as well as constraining short polymers at large distances and this is reflected by the ΔG^*^ trend upon elongation. ΔG^*^ decreases with the chain elongation for small binding angles while it increases for angles larger than 100°. This latter statement can be further appreciated by projecting the results on the angle/*N* plane (Fig. 4c), where it is also evident that the chelating effect reaches it maximum for chains with *N* < 25 and angles between 60° and 80°.

## Discussion and Conclusions

In summary, we have shown by a simple model that the chelation effect exists in flexible linkers of size comparable to that of the their target. While this result might appear intuitive, the effect is counterbalanced by the restriction in conformational space due to the excluded volume. These are the two effects contributing to the dissociation free energy: the excluded volume of the target and the length of the chain connecting the binders. The effect of the excluded volume is that of destabilizing the system at higher temperature and can be controlled by changing the chelating angle. The polymer length affects its end-to-end distance, directly related to the entropy. When the distance of the bindentate binder anchoring points corresponds to the average end-to-end distance of the free polymer, the conformational restriction is at its minimum, and the entropic conformational loss is minimized too.

While the polymer bulk concentration is a relevant parameter to estimate the experimental binding affinity, here we were interested in measuring the free energy associated with the polymer detachment at infinite dilution. The difference between bound and unbound state depends on their volume, as increasing the volume the unbound state increases its entropy whereas that of the bound state is unaffected, with an effect on the transition temperatures. For polymers adsorbed on a flat surface and for dilute systems, the surface coverage is known to be proportional to the polymer concentration reaching logarithmically a plateau at higher concentrations^30^. However, while shrinking the box in the explored case would simply keep the polymer bound to its target at higher temperatures, in real systems composed of thousand of coupled binders attempting to capture a number of particles in solution we would expect the formation of cross linked structures to compete with chelation with density fluctuations playing an important role^31^. While the study of interplay between chelation and cross linking at different thermodynamic conditions is an interesting issue in itself, it is beyond the scope of the presented work.

Here we have found that coupling two binding moieties through long polymeric flexible linkers is advantageous for a wide range of set-ups. As a linker affects positively the binding, the affinity of two weak binder will increase non linearly by coupling and selectivity will improve by targeting two non-equivalent binding sites which are unlikely to exist both on a different protein. This information can be readily exploited for the design of new nanodevices and new selective drugs.

## Additional Information

**How to cite this article**: Fortuna, S. *et al.* Chelating effect in short polymers for the design of bidentate binders of increased affinity and selectivity. *Sci. Rep.* **5**, 15633; doi: 10.1038/srep15633 (2015).
